# A Child Health Index for Sweden’s 290 Municipalities: A System of Indicators and Indices for Monitoring Children’s Health on the Local Level

**DOI:** 10.1007/s12187-017-9515-2

**Published:** 2018-01-15

**Authors:** Lennart Köhler, Bo Eriksson

**Affiliations:** 1 Nordic School of Public Health, Gothenburg, Sweden; 2 Epidemiology and Social Medicine, Department of Public Health, Institute of Medicine, The Sahlgrenska Academy at University of Gothenburg, Gothenburg, Sweden; 3 Health Metrics, Sahlgrenska Academy at University of Gothenburg, Gothenburg, Sweden

**Keywords:** Child health and wellbeing, Monitoring, Indicators, Index, Municipalities, Sweden

## Abstract

This broad survey of children’s health and wellbeing in Sweden’s 290 municipalities converts freely available national data to a set of 13 high quality indicators, and makes local surveillance and comparisons possible. Combining the indicators, using equal weights, into relevant domains as 5 sub-indices and then again into one summary index provides one index for the great picture, sub-indices for the various domains of child health and separate indicators for the detailed study of the basic components. This creates a simplified tool for decision makers and professionals in their task to monitor children’s health on the local level. Children’s health in the Swedish municipalities is generally good, with a mean Child Health Index of 88 out of 100, ranging from 81 to 93. Children in economically disadvantaged municipalities have, with few exceptions, more health problems and worse preconditions for health. The indicators Socio-economic standard, Tobacco in utero, Smoking households and Teenage abortions explain most of the municipality variations. But the broader range of indicators gives more information and is a better tool to consider strengths and weaknesses for each municipality, and is thus more useful for policy-oriented efforts. The real value of this kind of monitoring lies in a succession of comparable surveys. The generous, free and easily available data are not available in all other countries, but matters such as philosophy and design, indicator definitions and index constructions might be considered in other regions looking for ways to monitor children’s health and wellbeing on local levels.

## Introduction

In Sweden, as in many other countries, children’s health and well-being is a politically prioritized goal for Public Health activities. Since major parts of the social, educational and health promotional services are provided locally, the municipalities play an important role for the inhabitants’ welfare, not least for the children and their families. Thus, to be able to fulfill their tasks, the municipalities need a broad and actual knowledge of children’s living conditions. This knowledge will be used as a tool in practical and planning situations by professionals and policy makers, and should be well structured, easy to understand and possible to follow up to monitor the effects of interventions.

The use of indicators and indices, although always representing a simplification of reality**,** are therefore an attractive means for this purpose. In most sets of wellbeing indicators presented by international bodies such as WHO (World Health Organization), UNICEF (United Nations Children’s Fund), OECD (The Organization for Economic Co-operation and Development), EU (European Union), and Save the Children, data from assumed representative samples of large child populations are used and generalized in order to reflect and compare the situation in a country or a region as a whole. Aggregated data on the national level, however, cannot always be broken down sub-nationally, because they are often incomplete, not up-to-date, or simply not available. Therefore, special care must be taken to identify, collect and assess indicators on the municipal level. Although the goal is to create a practical tool, the information used to construct the indicators must be relevant, valid and available from reliable sources.

A useful way to cover the needs of relevant monitoring is to use a child public health approach, i.e. applying a broad definition of health, a population perspective, and a special consideration of children’s needs and conditions (Köhler 1998; Petticrew and Roberts 2004; Blair et al. 2010)*.*

Based on these conceptions some local indicator studies have been conducted in Scandinavia (Köhler 2006; Niclasen 2009). And commissioned by local politicians in the Swedish city of Gothenburg, other studies have recently been performed and presented as Child Health Indices for children 0–17 years, one for the most disadvantaged parts of the city (Köhler 2010) one for all parts of the city (Köhler 2013) and one for the surrounding region (Köhler and Henriksson 2013). Inspired by the results of these local reports the Swedish Association of Local Authorities and Regions (SKL) requested a similar analysis for all of Sweden’s 290 municipalities.

## Background

The purpose of the report prepared for SKL was to create a Child Health Index for all 290 municipalities in Sweden. The main objective was to distill and focus the abundant mass of data available on the national level, to be able to use them as a tool for decision makers and professionals to monitor children’s health and well-being locally. To map children’s health on the local level and compare the municipalities was also appealing, since differences between municipalities could stimulate local competition and act as an incentive for change and development. This report was published in Swedish in 2014 (Köhler 2014). The aim of the present paper is to present a condensed version with the statistical analyses focusing on the correlation structure of the indicators, domains and the population characteristics.

## Methods

To update the knowledge of children’s health and well-being in Sweden available publications were scrutinized, both scientific papers and local reports. Interviews were performed with representatives from national agencies within health and social affairs as well as with leading professionals in the child health and social sectors. Existing registers, mainly national data-bases, were searched for current information. For the age-group 0–17 years 13 health indicators were identified, which satisfied the need for relevance, quality and availability (Table 1). Latest available data (from 2012) were collected as percentages of the populations fulfilling the definition of the actual indicator. Some indicators reflect infrequent occasions, and to minimize temporary fluctuations data for 3 years were calculated, if possible. Due to lack of available data it has not been possible to separate results for boys and girls in a systematic way.

**Table 1:** Indicators and indices used for all 290 Swedish municipalities

Sub-index 1
*A. Socio-economy*
1. Children in poverty (children living in households with low national income standard or in households with means tested social assistance)
2. Pupils leaving compulsary school not elegible for higher studies
Sub-index 2
*B. Health and well-being*
1. Children hospitalized for external injuries
2. Children with mental health problems (national survey)
Sub-index 3
*C 1. Determinants - Risk-factors*
1. Low birthweight (under 2500 g)
2. Children exposed to tobacco in utero (mothers smoking)
3. Infants in smoking households
4. Adolescents daily smoking
5. Adolescents heavy drinking
6. Teenage abortions
Sub-index 4
*C 2. Determinants - Protective factors*
1. Children breastfed for 4 months
2. Children vaccinated against measles, mumps and morbilli (MPR)
Sub-index 5
*D. Service and support*
1. Children attending preschool
Summary Composite index
Sub-indices 1–5 added and divided by 5

Detailed definitions of included and refuted indicators with tables and figures for all 290 municipalities are available in Swedish (Köhler 2014, http://www.barnhalsoindex.se/index.php)

Information on another three population variables were also collected. They were, however, not included in the set of indicators, since they characterize the municipalities’ child populations and do not represent explicit determinants of health (Table 2).

**Table 2:** Child population characteristics (variables not included in the indices)

1. Number of children 0–17 years in the municipality
2. Child population as percentage of children 0–17 years in the population
3. Children of foreign origin (child and/or both parents born outside Sweden) as percentage of children 0–17 years in the population

Data satisfying scientific requirements for all indicators and background variables were available for each of the 290 municipalities in the country.

The indicators were structured into the following four domains, based on analyses adopted by the EU project Child Health Indicators for Life and Development (CHILD) (Rigby et al. 2003). These domains have also been used for Nordic systems of municipal level indicators (Köhler 2006, 2010, 2013, 2014; Niclasen 2009).

A) Demography and socio-economy
B) Health status and wellbeing
C) Determinants with two sub-domains: C1 risk factors and C2 protective factors
D) Service, support and health policy

Results will be presented for the domains as sub-index A, B, C1, C2, and D, plus a total Child Health Index (CHI).

The data of each indicator were transformed into a scale of 0–100, all defined in the same direction with higher figures meaning more favorable health or health determinant. Thus, if 60. 9% of the children were breastfed for 4 months the value of the indicator is 60.9, while if 10.3% of the infants were living in smoking households the value of that indicator will be 100 minus 10.3, i.e. 89.7.

To create a domain sub-index the indicators in the domain were added and the sum divided by the number of indicators, thus giving each indicator equal weight. The CHI was calculated as the unweighted mean of the five sub-indices.

In summary, the higher value of an indicator, the more favourable is the health/health determinant, and the higher value of a combined index, the more favourable are the conditions for the children in the municipality.

Means, standard deviations and correlations between indicators, domain sub-indices and the CHI were estimated using standard methods. Pearson correlations were used in spite of the slight, but systematic, negative skewness in the indicator distributions. The results obtained when applying non-parametric estimations did not deviate much. The correlations between indicators, sub-indices and the total index on the one hand and the municipality characteristics were studied, also using ordinary Pearson correlation and multiple linear regressions. To further examine the correlation structure of the indicators an explorative factor analysis was performed. The software used for analysis was Stata version 14.

The municipalities were divided into ten groups on the basis of structural parameters such as population, commuting patterns, tourism, industry and economic structure, according to the official Classification of Swedish municipalities (Table 3) (SKL 2011).

**Table 3:** Classification of Swedish municipalities, 2011

The following classification of Swedish municipalities is made by the Swedish Association of Local Authorities and Regions. The municipalities are divided into ten groups on the basis of structural parameters such as population, commuting patterns, tourism and travel industry and economic structure.
*1. Metropolitan municipalities* (3 municipalities)
Municipalities with a population of over 200,000 inhabitants.
*2. Suburban municipalities* (38 municipalities)
Municipalities where more than 50% of the night population commutes to work in another municipality. The most common commuting destination must be one of the metropolitan municipalities.
*3. Large cities* (31 municipalities)
Municipalities with 50,000–200,000 inhabitants and more than 70% of the population lives in urban areas.
*4. Suburban municipalities to large cities* (22 municipalities)
Municipalities in which more than 50% of the night population commutes to work in a large city.
*5. Commuter municipalities* (51 municipalities)
Municipalities in which more than 40% of the night population commutes to work in another municipality.
*6. Tourism and travel industry municipalities* (20 municipalities)
Municipalities where the number of guest nights in hotels, youth hostels and camping sites is higher then 21 nights per inhabitant and the number of holiday homes is higher then 0.20 per inhabitant.
*7. Manufacturing municipalities* (54 municipalities)
Municipalities where more than 34% of the night population aged 16 to 64 is employed in manufacturing, mining, energy, environmental and construction industries.
*8. Sparsely populated municipalities* (20 municipalities)
Municipalities where less than 70% of the population lives in urban areas and less than eight inhabitants per km^2.^
*9. Municipalities in densely populated regions* (35 municipalities)
Municipalities with more than 300,000 inhabitants within a 112.5 km radius.
*10. Municipalities in sparsely populated regions* (16 municipalities)
Municipalities with less than 300,000 inhabitants within a 112.5 km radius

## Results

Children’s health and living conditions were generally very good in the country. The mean Child Health Index of all municipalities was 88, ranging from 81 to 93 on the scale from 0 to 100 (Table 4).

**Table 4:** Statistics describing indicator distributions for 290 Swedish municipalities

			Mean	Standard deviation	Smallest value	Largest value	Range	Skewness
Indicators	A	Child poverty	88.2	4.13	67	96	29	−.87
		Pupils leaving school early	87.7	5.47	71	100	29	−.33
	B	Injuries	98.9	0.24	98	100	2	−.33
		Mental health problems	86.3	2.80	76	97	21	−.05
	C1	Low birth weight	98.1	0.83	96	100	4	−.40
		Tobacco in utero	89.4	2.43	81	94	13	−.63
		Infants in smoking households	71.7	7.90	48	93	45	−.28
		Adolescents daily smoking	96.8	3.05	74	100	26	−2.90
		Adolescents heavy drinking	96.9	0.90	94	100	6	−.11
		Teenage abortions	91.5	3.53	77	99	22	−.56
	C2	Breastfeeding	90.6	3.48	73	98	25	−1.12
		Vaccinations	74.9	7.89	51	94	43	−.34
	D	Service and support (attending preschool)	83.5	4.99	66	98	32	−.76
		Name	Mean	Standard deviation	Smallest value	Largest value	Range	Skewness
Domain indices	A	Socio-economy	87.9	4.06	73	97	24	−.43
	B	Health and well-being	92.6	1.43	87	98	11	−.11
	C1	Risk factors	90.7	2.00	83	95	12	−.45
	C2	Protective factors	84.3	4.18	64	100	36	−.50
	D	Service and support	83.5	4.99	66	98	35	−.76
Child health index			87.8	2.10	81	93	12	−.12

**Fig. 1:**
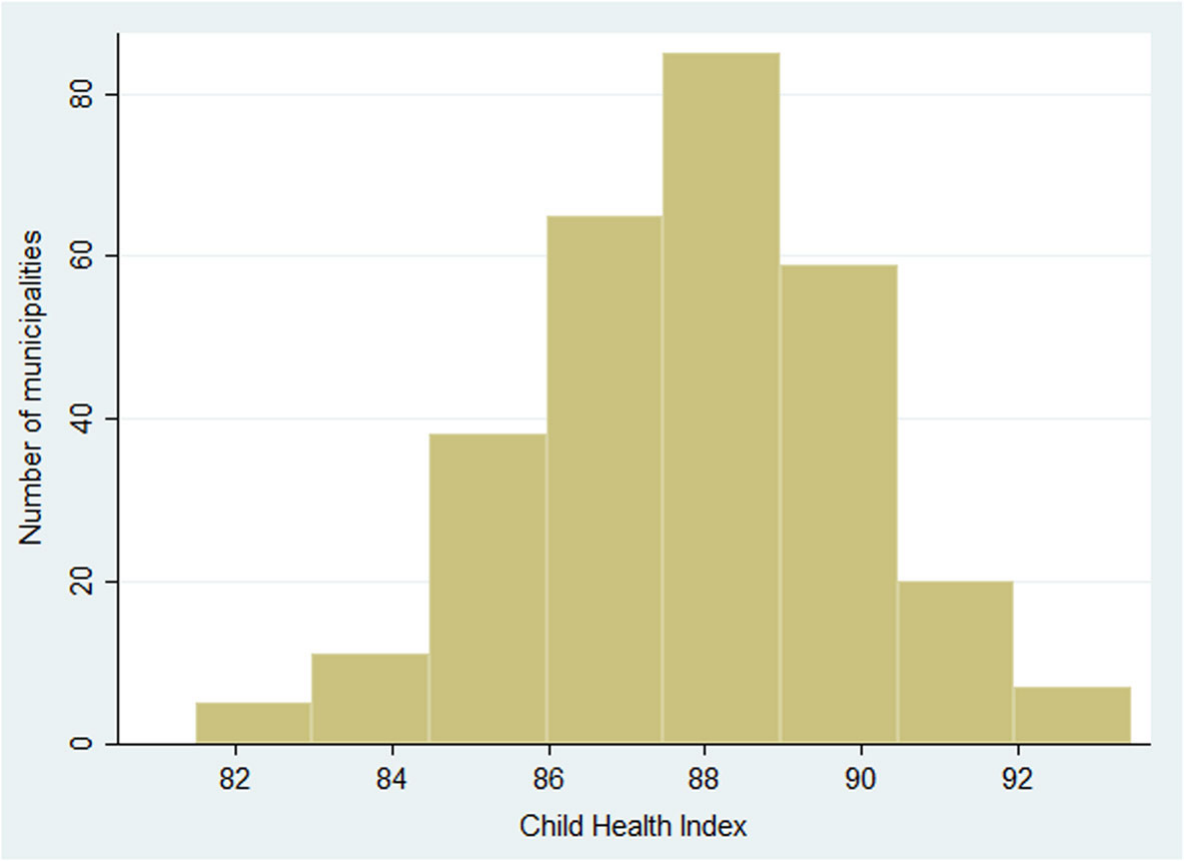
Distribution of child health index in Sweden’s 290 municipalities 2012

The distribution is close to symmetric. The individual indicator distributions are negatively skewed. Taking the mean brings the distribution closer to symmetry, as expected from statistical theory (Fig. 1).

For Child poverty the indicator values, on the scale 0–100, varied between 67 (highest level of poverty) and 96 (lowest level of poverty) i.e. 29% units.

In Domain B, Health and Well-being, the indicator values varied greatly between the municipalities. The incidence of External injuries was more than three times higher in some municipalities than in some others. Mental health problems were generally less frequently reported in small municipalities with low prevalence of Childhood Poverty and few children with immigrant background. But there were single exceptions.

In Domain C1 the Risk-factors Low birth weight, Tobacco in utero, Smoking in infant families were more common among economically disadvantaged municipalities. Some indicators reflect rather seldom occurring health problems, but the difference between the municipalities may be great. Thus, both Low birth weight and Teenage abortions were infrequent happenings, but the ratios between highest and lowest incidence were 6 and 20, respectively.

The C2 Protective indicators showed generally good levels, with particularly high coverage of Vaccination, with a mean of 96 and more than 10% of the municipalities having 100% of their children vaccinated. Also Breastfeeding for 4 months was high, with a mean of 76, but several municipalities did not reach 60.

In Domain D, Service and Support, only one indicator was available. Pre-school attendance of children 1–5 years is an offer which is compulsory for the municipalities but optional for the parents. The attendance, both in publicly and privately run preschools, was high, mean 84. A second indicator used in other local Swedish studies, Parents of infants participating in sessions at Well Baby Clinics, was not available in all municipalities.

**Fig. 2:**
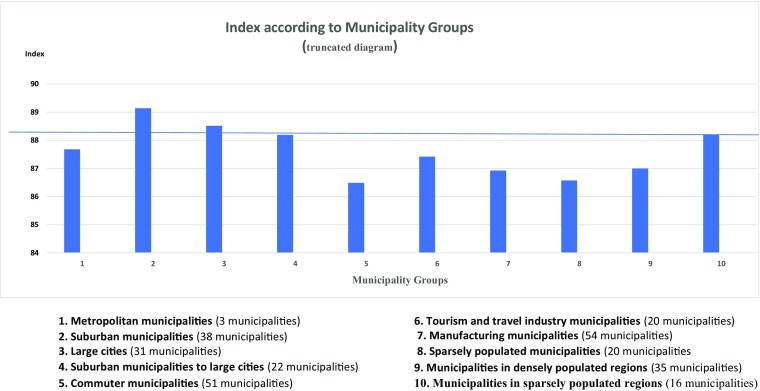
Mean child health index in the 10 municipality groups

The Child Health Index demonstrates, as expected, that municipalities with a low childhood poverty indicator generally had a low Index, and that economically advanced municipalities had the highest Index. There were a few small municipalities which did not follow this pattern by having poor economy and high indices or vice versa. There was no statistically significant correlation between the indicators or indices and the official classification groups of municipalities, defined by geographical localization, urban and rural situation and type of economic activities. However, municipalities with low Child Health Index were dominated by industry and agriculture, while those with a high Child Health Index were mainly suburban areas outside the big cities, some densely populated municipalities, and a few with very small populations (Fig. 2).

The distributions of the indicators are negatively skewed which is natural since 100% is the upward limit and some municipalities are drastically lower than others. The distributions of the domain sub-indices are less skewed. The total Child Health Index has a fairly symmetric distribution over the 290 municipalities (Fig. 1).

The demographic characteristics of the municipality child populations (Table 2), vary greatly. The big cities, Stockholm, Gothenburg and Malmö have 166,000, 98,000 and 59,000 children 0–17 years, respectively, while some of the smallest municipalities have less than 500 children. Also other demographic measures are different: in a few municipalities more than 45% of the children or both their parents were born outside Sweden, while in some other municipalities the corresponding figures were 2–3%, a range of 47% units. For the whole country 19% of the children had a foreign origin.

The correlations between the indicators are mainly weak or modest and generally positive, meaning that large values for one indicator most often correspond with large values for another indicator (Table 5). Correlations exceeding 0.11 are statistically significant at the 5% level. Child poverty, Tobacco in utero and Teenage abortions are highly correlated to 4 other indicators. At the other end, Injuries, Low birth weight, Heavy drinking and Service do no associate statistically significantly to any other indicator. The total correlation pattern is examined below in connection with the factor analysis.

**Table 5:** Pearson correlation coefficient between indicators

	Child poverty	Leaving school early	Injuries	Mental health problems	Low birth weight	Tobacco in utero	Infants in smoking households	Adolescents daily smoking	Adolescents heavy drinking	Teenage abortions	Breastfeeding	Vaccinations	Service and support (attending preschool)
Child poverty	1												
Leaving school early	**.42**	1											
Injuries	.07	−.01	1										
Mental health problems	.05	.06	**.18**	1									
Low birth weight	**.15**	.05	.06	**.14**	1								
Tobacco in utero	**.35**	**.23**	**.12**	**.18**	.06	1							
Infants in smoking households	**.28**	**.29**	.09	−.02	**−.13**	**.43**	1						
Adolescents daily smoking	.08	−.00	−.05	.09	.01	−.04	−.09	1					
Adolescents heavy drinking	.09	**.13**	.01	.06	−.02	**.17**	.11	.07	1				
Teenage abortions	**.45**	**.38**	**.12**	.02	.07	**.54**	**.50**	.02	**.23**	1			
Breastfeeding	**.15**	−.01	**.12**	**.37**	.03	**.39**	.04	.03	−.09	.10	1		
Vaccinations	−.08	−.07	.04	**.17**	−.02	**.39**	**.15**	−.02	−.01	.07	**.20**	1	
Service and support (attending preschool)	.03	.08	.10	**.16**	−.02	.10	.06	.02	−.03	.10	.00	.04	1

Correlations deviating more than 0.11 from zero are statistically significant at the 5% level and marked in bold

The correlation between the domains C1 and C2 is strong meaning that if a municipality has a high value for risk factors (risks are low) it has also a high value for protective factors (good protection) (Table 6).The comparatively high correlations between the social and demographic index and the risk and protective indices are expected considering the underlying indicator correlations seen in Table 4.

**Table 6:** Pearson correlation coefficients between domain indices

	A Socio-economy	B Health and well-being	C1 Risk factors	C2 Protective factors	D Service and support
A.Socio-economy	1				
B. Health and well-being	.07	1			
C1. Risk factors	**.46**	.07	1		
C2. Protective factors	**.34**	.02	**.88**	1	
D. Service and support	.07	**.16**	.09	.08	1

Correlations exceeding 0.11 are statistically significant at the 5% leveland marked in bold

By using an explorative factor analysis of a set of variables a smaller number of new variables, factors, can be defined, which contain most of the total variation for all the original variables. In this study the factor analysis of the 13 indicators leaves four factors to be considered. The first factor represents 61% of the total variation and the second one adds another 25%.

Indicators with high loadings in the first, and to some extent, the second factor should be considered important (Table 7). The first factor has fairly high loadings for the Socio-economic indicators, Tobacco in utero, Smoking households and Teenage abortions. The second factor is mainly Vaccination and again Tobacco in utero. The third factor reflects the Mental health problems, Breastfeeding and the Preschool indicator, but explains only a small portion of the total variation. The fourth factor explains almost no variation. Thus, the Socio-economic indicators, Tobacco in utero, Smoking in households and Teenage abortions emerge as the most informative indicators.

**Table 7:** Factor loadings and communalities estimated in the factor analysis of all indicators

		Factor 1 loading	Factor 2 loading	Factor 3 loading	Factor 4 loading	Communality %
A	Child poverty	.63				46
	Leaving school early	.55				31
B	Injuries			.25		11
	Mental health problems			.52		33
C1	Low birth weight				.24	11
	Tobacco in utero	.45	.80			87
	Infants in smoking households	.52	.25		−.27	43
	Adolescents daily smoking					7
	Adolescents heavy drinking	.24				15
	Teenage abortions	.73	.23			59
C2	Breastfeeding		.32	.50		39
	Vaccinations		.80			66
D	Service and support (attending preschool)			.21		9

Only factor loadings above .20 are shown

For the sub-indices the first factor represents more than 98% of the total variation and is the only factor of importance (Table 8). It is a weighted mean of A, C1 and C2 with the weights for C1 and C2 considerably higher than the weight for A. As expected high factor loadings at this level is also linked to high communality as is clearly seen in the table. The three domains A, C1 and C2 contain the indicators that came out as most important at the indicator level.

**Table 8:** Factor loadings and communalities estimated in the factor analysis of domain indices

Domain	Name	Factor 1 loading	Factor 2 loading	Communality %
A	Socio-economy	.45		25
B	Health and well-being		.31	10
C1	Risk factors	.92		87
C2	Protective factors	.90		82
D	Service and support		.28	10

Only factor loadings above .20 are shown

The results of Tables 7 and 8 takes the analysis of correlations of Tables 5 and 6 one step further, and show that indicators with high factor loadings in the first factor are most important.

Table 9 shows how the indicators and domain indices correlate to the three demographic municipality variables. Correlations of >0.12 or <−0.12 deviate significantly from zero at the 5% level. The correlations with population size and proportion of children show largely similar patterns over the indicators and domain indices. All these correlations are positive with few insignificant exceptions. The correlations with the variable indicating proportion of children of foreign origin are systematically negative, signalling disadvantage for the CHI. The strongest correlations, both positive and negative, appear for Child poverty, Injuries, Smoking in households, Teenage abortion and the domain indices C1 and C2.

**Table 9:** Simple Pearson correlations between indicators and three demographic background variables. The rightmost column shows the R-square values for the multiple linear regressions

		Correlation with municipality population size	Correlation with proportion of children 0–17	Correlation with proportion of children with foreign background	Multiple regression explained variance (R2) %
	Indicator				
A.	Children in poverty	**0.13**	**0.38**	**−0.54**	53
	Pupils leaving school early	0.04	**0.24**	**−0.31**	16
B.	Children hospitalized for injuries	**0.26**	**0.15**	**0.22**	10
	Children with mental health problems	0.06	0.01	0.03	0.1
	Low birth weight	0.02	0.02	−0.02	0.1
C 1.	Children exposed to tobacco in utero	**0.33**	**0.28**	**−0.12**	14
	Infants in smoking households	**0.25**	**0.28**	−0.11	15
	Adolescents daily smoking	0.04	0.04	−0.04	0.4
	Adolescents heavy alcohol drinking	−0.04	0.04	−0.09	1.3
	Teenage abortions	**0.32**	0.31	−0.15	20
C 2.	Children breastfed for 4 months	**0.14**	0.11	−0.02	1.4
	Children vaccinated against MPR	−0.01	0.02	0.03	0.6
D.	Attending preschool	0.07	0.04	0.01	0.6
	Domain indices				
	A Socio-economy	0.05	**0.35**	**−0.48**	41
	B Health and well-being	0.04	0.03	0.06	0.5
	C1 Risk factors	**0.25**	**0.33**	−0.11	22
	C2 Protective factors	0.19	0.25	−0.09	13
	D. Service and support	0.07	0.04	0.01	0.6
	Child health index	**0.14**	**0.32**	**−0.23**	23

Correlations of >0.12 or <−0.12 deviate statistically significantly from zero at the 5% level and marked in bold

The squared multiple correlation (R2) shows how much of the variation in indicators and indices that can be considered “explained” by the three population characteristics.

More than half (53%) of the child poverty variation can in this sense be attributed to the three variables, most important is the proportion of children with foreign background. The corresponding figure for domain index A is 40%, while indices C1 and C2 end somewhat lower. R2 over or near 50% should be considered as fairly strong.

**Fig. 3:**
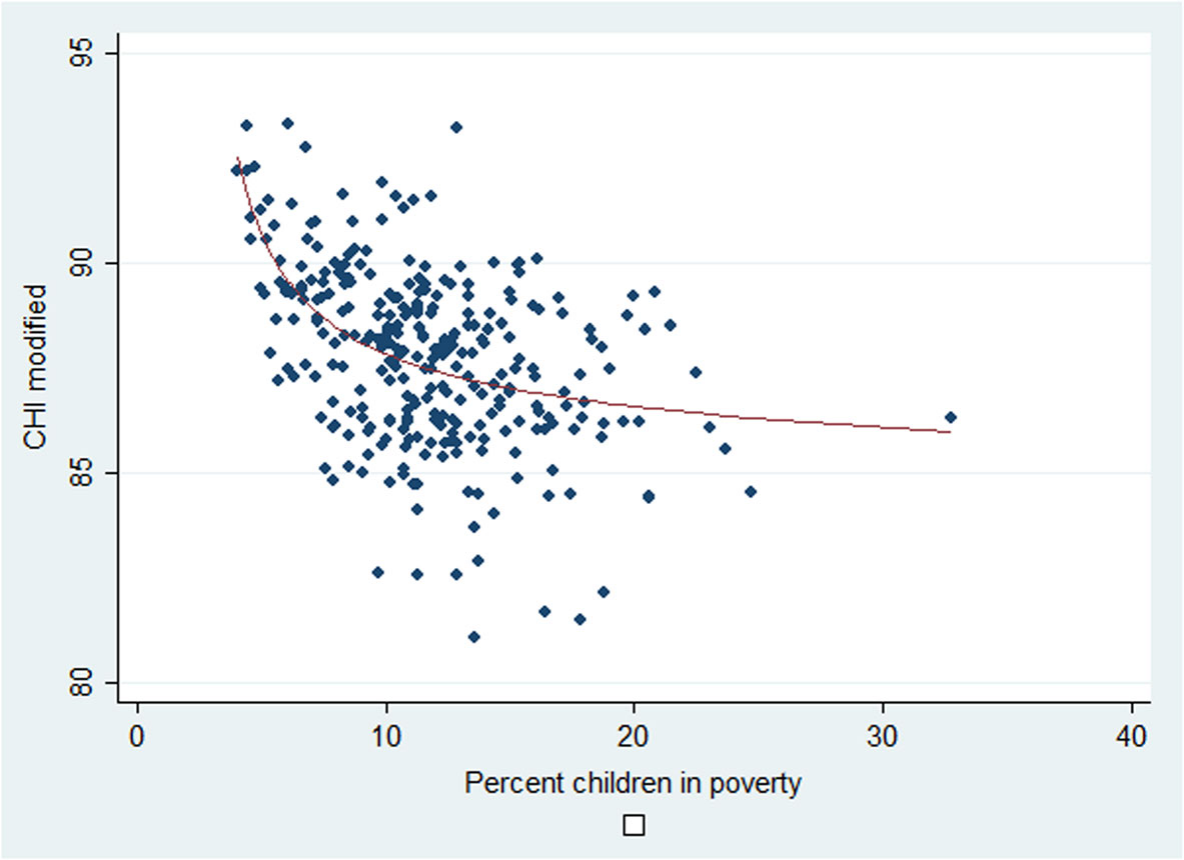
Scatter plot of the child health index against the percentage of children in poverty

Figure 3, showing the Child Health Index (without the poverty indicator) related to the poverty indicator, has two components, a scatter plot and a curve fitted using the fractional polynomial approach (Roystone and Altman 1994). The best fitting model is then a linear combination of the inverse of x squared and the logarithm for x. The Adjusted R-Square is 22.6%, not very high as is obvious also from the graph.

## Discussion

The aim of the original report was to present an updated and broad survey of children’s health and wellbeing in the Swedish municipalities. The goal was to increase the knowledge and to provide better opportunities for service and policy organizations to improve and promote children’s health and its determinants on the local level, an explicit policy perspective (Ben-Arieh 2008b). The content should be comprehensive and cover actual and important aspects. Thus, based on the well-established and regularly validated official registers that exist in Sweden, the set of 13 indicators fulfilled reasonably well the ambitions of relevance, quality and availability. The way of presenting the indicators in a common, uniform and comparable scale makes the results easy to grasp. Other methods have been used to simplify the results, such as ‘z scores’, which reveal how far the result for an area falls above or below the average for a group of areas (UNICEF 2007; Bradshaw et al*.* 2007). Since the main purpose here was not to compare the municipalities, but to present an easy way to monitor the health and wellbeing of children on the local level, the use of these kind of calculations did not seem to add important knowledge.

Although the pros and cons of a composite index is vividly debated (Jacobs et al. 2006; OECD 2008; Ben-Arieh 2008a; Köhler 2016) it is widely considered to summarize the complex process of monitoring health, facilitating comparisons between areas and periods, and also making it more accessible for advocacy and interventions, both for decision makers and professionals (UNICEF 2007; Bradshaw et al*.* 2007; Heshmati et al*.* 2007; Dijkstra 2009; MacKay and Vincenten 2012; Hagerty and Land 2007, 2012; Köhler 2016). Most indicator systems in operation use an equal weighting system since differential weighting seldom yield different conclusions, and a good method for determining and allocating appropriate weights has not been identified (Bradshaw et al. 2007; Hagerty and Land 2004, 2007, 2012).

Some indicators which carry important information could not be used, since they were not properly registered in all municipalities (e.g. dental caries, overweight/obesity) or were inconsistently defined or applied (e.g. children with asthma, children placed in municipal care or in special education) or had a too low prevalence to be broken down to local levels (e.g. infant mortality, suicide).

The final 13 indicators provided an acceptable coverage of the health area and for its purpose seem to be the best attainable for the time being. But there are still important parts of the child population and its health panorama which are not covered. The indicators, thus, cover reasonably well newborns and schoolchildren, but much less so preschool children. This is a critical deficit since we need appropriate knowledge about this period to be able to invest wisely in children’s early development. Then there is a total lack of systematic data on some health topics, such as children’s nutritional habits and physical activity, their abuse, neglect or intentional injuries, their long term disabilities, their social cohesion or social capital, their own appreciation of their health and health problems, the threats and support of their physical, social and mental environment, their cultural experiences, just to mention some of the increasingly important areas of health and its determinants.

Although gender is strongly recommended to be considered in all data collections it was too seldom taken into account.

A previous study in Swedish municipalities using the same criteria could in 1990 identify 5 indicators, in 2000 6 indicators (Köhler 2006) and now in 2012 13 indicators. There are several continuing efforts to improve the data collection of children’s health and well-being in Sweden, and in a few years it will undoubtedly be possible to identify additional important indicators and indices. Then, hopefully, also reliable data of the more or less invisible marginalized children will be collected, e.g. children in State Care, children of minority ethnic groups and the unprecedented number of children fleeing to Sweden to avoid war and persecution in other parts of the world. All these children have excessive needs of care and support of various kinds, not least on the local level.

In the early 1990’s a national public health report summarized the actual situation in Sweden: *“One looks in vain for a systematic, continuous and complete reporting of children’s health, in a child’s perspective and related to a social context”* (Köhler and Jakobsson 1991). This conclusion is still valid.

A detailed description, definition and sources of indicators used and discarded are given in previous publications (Köhler 2006 in English, and Köhler 2014 in Swedish with a summary in English).

As expected this study showed a bright picture of children’s health and wellbeing in Sweden, with a high Child Health Index and a rather narrow range between the best and the worst municipality. Nonetheless, for a few indicators there were apparent variations, particularly for risk indicators, such as Low birth weight and Teenage abortions, just as there were differences in size and other demographic characteristics. On the other hand, an indicator such as Vaccination showed very small variation between the municipalities. Also as expected, municipalities with high childhood poverty generally had a low Child Health Index, and economically advanced municipalities had the highest Index, although there were also here a few exceptions.

Since the main goal of the study was to create a tool for monitoring children’s health and wellbeing, and not comparing the municipalities, the statistical analyses focused on correlation structures between the indicators and indices and relating them to the three population variables, characterizing the social and economic background of the children, and, further, relating them to the official classification of municipalities, defined by geographical localization, urban and rural situation and type of economic activities. To develop other spatial dimensions was not considered to be relevant for the goal.

The analysis showed that a few indicators, Child poverty, Tobacco in utero, Teenage abortions, had a high correlation with other indicators. The two Socio-economic indicators, Tobacco in utero, Smoking households and Teenage abortions came out as most important at the indicator level and the three domains A, C1 and C2 as most important on the domain level. At the other end a few indicators, Mental health problems, Breastfeeding, Preschool attendance, explained only a small portion of the total variation.

The population characteristics of the municipality were important factors for the level of the Child Health Index, as well as for most of the sub-indices and several indicators. Generally, the correlations to population size and the percentage of children 0–17 years in the population were positive, meaning that bigger municipalities and more children often predicted higher indicator values. On the other hand, the correlations to the proportion of children with foreign background were mainly negative, i.e. higher proportions tend to mean lower indicator values. For example, municipalities with large proportions of children with foreign background had statistically significantly more often low levels of the poverty and tobacco environment indicators. The strongest correlations for all the population characteristics were the ones with a high proportion of children in poverty and leaving school early.

The areas studied were the administrative units of ‘municipality’, which however, vary quite a lot in size, demography and socio-economic conditions. Particularly the three big cities show variations within the municipalities, which can be larger than the variation between the municipalities (Köhler 2013). For better understanding and more effective interventions the big municipalities should preferably be broken down into smaller parts, as was previously done for the second biggest city, Gothenburg (Köhler 2010, 2013). The official classification of the municipalities in 10 groups according to some structural parameters did not help explaining the outcomes of the indicators or indices.

To measure and compare the health status of a municipality the use of 5 or even 4 indicators could be argued to give enough information, particularly if the resources for monitoring are scarce. On the other hand, the addition of the remaining indicators provides a broader and more detailed panorama of children’s health and its determinants and gives professionals and decision makers better opportunities to consider strengths and weaknesses for each municipality and to act accordingly. So, for instance, even if Breastfeeding and Vaccination do not explain much of the variation among the municipalities, it is valuable to be able watch that the high levels are maintained everywhere. Besides, the costs of adding a few more indicators are negligible, since all data are easily and freely available. The actual problem is not to have too many relevant indicators, but not being able to cover the obvious gaps in important areas of children’s health panorama and to include also especially vulnerable groups of the child population. Particularly urgent seems to cover areas such as overweight/obesity and dental decay. Substantial knowledge exists about these highly prevalent and genuine health problems on the national level and also in some counties and municipalities, but systematic data are missing on the municipality level.

A single indicator survey, no matter how sophisticated and elaborated, gives only a snapshot of the actual situation. The real value of this kind of monitoring, appears first when a succession of comparable surveys are performed. They will then act as a powerful local and comparative tool, making it possible for professionals to follow the development of children’s health and its determinants, for policy makers to stress the importance of children and their well-being, for politicians to keep abreast with the situation in their areas of responsibility and to monitor the effects of their policy interventions.

The generalization of this study is limited. Every country or region is unique and must be considered as such. In that sense, the study is strictly contextual. Sweden is a highly developed country with generous, free and easily available information of society structures, not least of health and health care. Much of the information used here is simply not available in other countries. But there are certainly elements in the study that can be picked up, transformed and modified for use in other contexts, such as the study philosophy and design, indicator definitions, index constructions.

## Conclusion

This Child Health Index for the 290 Swedish municipalities is based on a Child Public Health approach. By identifying 13 indicators from freely available national data bases and combining them into 5 sub-indices and one summary Child Health Index a well-structured, easy to understand and practical tool is offered to decision makers and professionals, helping them to identify where best to follow-up, improve and promote children’s health and its determinants on the local level. The indicators Socio-economic standard, Tobacco in utero, Smoking households and Teenage abortions explain most of the variations and could very well be used for a brief comparison and ranging of the municipalities. However, a broader range of indicators gives more information and is a better tool to consider strengths and weaknesses for each municipality, and is thus more useful for policy-oriented efforts. The real problem is not too many relevant indicators, but the obvious gaps in important areas of children’s health, particularly for vulnerable children.

The indicators and the combined indices mirror a generally good health among children, but also show clearly that children in economically disadvantaged municipalities, with few exceptions, have more health problems and worse preconditions for health. The highest Child Health Index is generally found in suburban areas outside the big cities, in some densely populated municipalities, and in a few with very small populations. Low Child Health Index municipalities are dominated by industry and agriculture, and with a high proportion of children with foreign background. A survey like this one should be repeated with certain intervals, to serve as a reliable tool for monitoring children’s health and well-being on the local level.

The generous, free and easily available data are not available in all other countries, but matters such as philosophy and design, indicator definitions and index constructions might be considered in other regions looking for ways to monitor children’s health and wellbeing on local levels.

## References

[CR1] Ben-Arieh A (2008). The child indicators movement: past, present and future. Child Indicators Research.

[CR2] Ben-Arieh A (2008). Indicators and indices of children’s well-being: toward a more policy oriented perspective. European Journal of Education.

[CR3] Blair M, Stewart-Brown S, Waterston T, Crowther R (2010). Child public health.

[CR4] Bradshaw J, Hoelscher P, Richardson D (2007). An index of child well-being in the European union. Journal of Social Indicators Research.

[CR5] Dijkstra T (2009). Child well-being in rich countries: UNICEF’s ranking revisited, and new symmetric aggregating operators exemplified. Child Indicators Research.

[CR6] Hagerty, M. R., & Land, K. C. (2004). Constructing summary indices of social well-being. A model for the effect of heterogeneous importance weights. University of California, Davis and Duke University.

[CR7] Hagerty MR, Land KC (2007). Constructing summary indices of quality of life: a model for the effect of heterogeneous importance weights. Sociological Methods & Research.

[CR8] Hagerty MR, Land KC, Land KC (2012). Issues in composite indicators construction. The well-being of America's children: Developing and improving the child and youth well-being index. Children’s well-being: indicators and research. Chapter 6.

[CR9] Heshmati, A., Bajalan, C., & Tausch, A. (2007). *Measurement and analysis of child well-being in middle and high income countries*. IZA Document Paper, No. 3203, Institute for the Study of Labor, Bonn.

[CR10] Jacobs, R., Goddard, M., & Smith, P. S. (2006). *Public services: Are composite measures a robust reflection of performance in the public sector?* Research Paper, Centre for Health Economics, University of York.

[CR11] Köhler L, Jakobsson G (1991). Children’s health in Sweden. An overview for the 1991 Public Health Report.

[CR12] Köhler L (1998). Child public health: a new basis for child health workers. European Journal of Public Health.

[CR13] Köhler L (2006). Health indicators for Swedish children. A contribution to a municipal index.

[CR14] Köhler, L. (2010). *Barnhälsoindex för stadsdelarna i nordöstra Göteborg. (A Child Health Index for the North-eastern parts of Gothenburg. A system for monitoring children’s health and well-being)*. NHV-Report 2010:3. Gothenburg, Nordic School of Public Health.

[CR15] Köhler, L. (2013). *Barnhälsoindex för Göteborg. Ett system för att följa barns hälsa i Göteborg och dess stadsdelar. (A Child Health Index for the city of Gothenburg and its town districts. A system of indicators for monitoring children’s health)*. NHV-Report 2013:3 R. Gothenburg, Nordic School of Public Health.

[CR16] Köhler, L., & Henriksson, G. (2013). *Barnhälsoindex för Västra Götalandsregionen. Ett system för att följa barns hälsa i Västra Götalandsregionen och dess kommuner. (Child Health Index for the 49 municipalities in the Region of Vastra Gotaland in South-West of Sweden. A system of indicators for monitoring children’s health)* NHV-Report 2013:5 R. Gothenburg, Nordic School of Public Health.

[CR17] Köhler L (2014). A child health index for Sweden’s 290 municipalities. A system of indicators for monitoring children’s health.

[CR18] Köhler L (2016). Monitoring children’s health and well-being by indicators and index: apples and oranges or fruit salad?. Child: Care, Health and Development.

[CR19] MacKay M, Vincenten J (2012). Child safety report card: How safety conscious are European countries towards children? Europe summary for 31 countries.

[CR20] Niclasen, B. (2009). *Indicators on children’s health and wellbeing in Greenland*. Doctoral thesis. Gothenburg, Nordic School of Public Health.

[CR21] OECD (2008). Handbook on constructing composite indicators: Methodology and user guide.

[CR22] Petticrew M, Roberts H (2004). Child public health and social welfare: lessons from the evidence. Child: Care, Health and Development.

[CR23] Rigby M. J. (2003). Child Health Indicators for Europe: A priority for a caring society. The European Journal of Public Health.

[CR24] Roystone P, Altman DG (1994). Regression using fractional polynomials of continuous variables: parsimonious parametric modelling. JRSS.

[CR25] SKL. (2011) Classification of Swedish municipalities https://skl.se/tjanster/englishpages.411.html.

[CR26] UNICEF. (2007). *Child poverty in perspective: An overview of child well-being in rich countries*. Florence, Innocenti Report Card 7.

